# MR bone imaging

**DOI:** 10.1186/2050-5736-3-S1-O37

**Published:** 2015-06-30

**Authors:** Wilson Miller

**Affiliations:** 1 University of Virginia, Charlottesville, Virginia, United States

## Background/introduction

Bone is highly relevant to focused ultrasound therapy, both as a potential treatment target and because it interferes with treatment of other organs such as the brain. It is challenging to image cortical bone using MRI, however, due to low water density and fast signal decay in bony tissues. Ultrashort echo time (UTE) imaging is a specialized MR technique that allows the weak, short-lived signal from cortical bone to be imaged despite these limitations. Potential applications of UTE bone imaging in MR-guided focused ultrasound include direct MR thermometry of bone heating, which is not possible using standard proton resonance shift (PRFS) techniques, and *in situ* skull imaging during brain treatment procedures, which could replace the separate CT scan currently required for transcranial focused ultrasound.

## Methods

In UTE MRI, imaging data is acquired using a spoke-radial k-space trajectory with gradient ramp sampling. This allows data acquisition to begin immediately after RF excitation, to capture the MR signal from cortical bone before it decays away. The basic UTE imaging technique can be implemented as either a 2D slice-selective or a 3D volumetric acquisition. A volumetric acquisition is ideal for 3D skull imaging. Achieving adequate spatial resolution, however, requires a relatively long scan time (~10 min). Fast UTE imaging of 2D slices can be performed by using specially designed half-RF pulses. Although more challenging to implement, such an acquisition would be much more suitable for monitoring transient temperature changes in bone during FUS treatment. Because bone signal decays too quickly to perform PRFS-based thermometry, however, other temperature-dependent MR properties must be used to generate sensitivity to temperature changes. For instance, the T1 relaxation time generally decreases with increasing temperature, whereas T2 generally increases. Either of these effects might therefore provide a basis for MR thermometry in cortical bone.

## Results and conclusions

**Figure 1:**
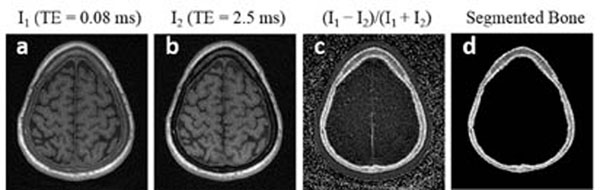
MR images reconstructed from a dual-echo 3D scan of a human head, showing the potential for skull imaging and segmentation using UTE MRI. Imaging resolution is 1.25 mm isotropic.

**Figure 2:**
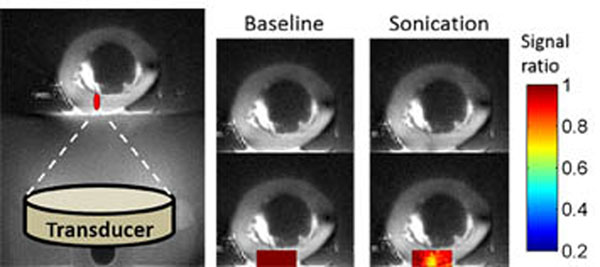
T1-weighted UTE images of a beef long bone during focused ultrasound application, showing the potential for MR thermometry using UTE MRI. To make the technique quantitative, the size of the T1-dependent signal decrease must be calibrated to temperature.

Figure1 shows several images of the same 1.25mm-thick slice, reconstructed from a dual-echo 3D UTE head scan. Cortical bone in the skull has weak, but clearly nonzero, signal in the UTE image (a), but appears black in the long-TE image (b). The conspicuity of cortical bone can be enhanced by taking the difference of these images and dividing by their sum (c), which facilitates automatic segmentation of bone pixels (d). Investigations are currently underway to use such UTE bone scans in place of CT scans to compute aberration corrections for transcranial ultrasound. Figure 2 shows T1-weighted UTE images of a beef bone obtained during focused ultrasound heating. The MR signal decreases at the location of focal heating, which is the expected behavior as T1 increases. Calibrating such signal changes to quantitative temperature changes is the focus of ongoing research. In conclusion, MR bone imaging using UTE techniques holds substantial promise for improving MR-guided focused ultrasound treatments of bone and other organs. Although UTE pulse sequences are not standardly available on existing commercial scanners, this situation is likely to change over the next few years.

